# Medical records and issues in negligence

**DOI:** 10.4103/0970-1591.56208

**Published:** 2009

**Authors:** Joseph Thomas

**Affiliations:** Department of Urology, Kasturba Medical College, Manipal 576 104, India

**Keywords:** Medical records, medical negligence

## Abstract

It is very important for the treating doctor to properly document the management of a patient under his care. Medical record keeping has evolved into a science of itself. This will be the only way for the doctor to prove that the treatment was carried out properly. Moreover, it will also be of immense help in the scientific evaluation and review of patient management issues. Medical records form an important part of the management of a patient. It is important for the doctors and medical establishments to properly maintain the records of patients for two important reasons. The first one is that it will help them in the scientific evaluation of their patient profile, helping in analyzing the treatment results, and to plan treatment protocols. It also helps in planning governmental strategies for future medical care. But of equal importance in the present setting is in the issue of alleged medical negligence. The legal system relies mainly on documentary evidence in a situation where medical negligence is alleged by the patient or the relatives. In an accusation of negligence, this is very often the most important evidence deciding on the sentencing or acquittal of the doctor. With the increasing use of medical insurance for treatment, the insurance companies also require proper record keeping to prove the patient's demand for medical expenses. Improper record keeping can result in declining medical claims. It is disheartening to note that inspite of knowing the importance of proper record keeping it is still in a nascent stage in India. It is wise to remember that “Poor records mean poor defense, no records mean no defense”. Medical records include a variety of documentation of patient's history, clinical findings, diagnostic test results, preoperative care, operation notes, post operative care, and daily notes of a patient's progress and medications. A properly obtained consent will go a long way in proving that the procedures were conducted with the concurrence of the patient. A properly written operative note can protect a surgeon in case of alleged negligence due to operative complications. It is important that the prescription for drugs should be legible with the name of the patient, date, and the signature of the doctor. An undated prescription can land a doctor in trouble if the patient misuses it. There are also many records that are indirectly related to patient management such as accounts records, service records of the staff, and administrative records, which are also useful as evidences for litigation purposes. Medical recording needs the concerted effort of a number of people involved in patient care. The doctor is the prime person who has to oversee this process and is primarily responsible for history, physical examination, treatment plans, operative records, consent forms, medications used, referral papers, discharge records, and medical certificates. There should be proper recording of nursing care, laboratory data, reports of diagnostic evaluations, pharmacy records, and billing processes. This means that the paramedical and nursing staff also should be trained in proper maintenance of patient records. The medical scene in India extends from smaller clinics to large hospitals. Medical record keeping is a specialized area in bigger teaching and corporate hospitals with separate medical records officers handling these issues. However, it is yet to develop into a proper process in the large number of smaller clinics and hospitals that cater to a large section of the people in India.

## METHODS OF RECORD KEEPING

The traditional method of keeping records that is followed in most of the hospitals across India is the manual method involving papers and books. There are serious limitations of manual record keeping including the need for large storage areas and difficulties in the retrieval of records. However, it is legally more acceptable as a documentary evidence as it is difficult to tamper with the records without detection. The present era has seen the computerization of medical records that are neat and tidy, and can be easily stored and retrieved. However, the possibility of easy manipulation without detection is a serious concern; hence, they may not be universally accepted at face value as a documentary evidence. If it is demanded during court proceedings, it is the duty of the hospital and the doctor to prove that these computer documents were not altered. Another major concern is maintaining confidentiality of the patient records as the patient can hold the doctor and the hospital negligent for breaking confidentiality of his medical records. Video tapes of endoscopic procedures, electronic fetal heart monitor charts, continuous ECG or Pulse oximeter charts could become important evidence in a court of law. Electronic medical recording is in the process of evolution and is being increasingly used. Though the total avoidance of paper records is the ideal aim, there are many areas that need to be sorted out. For example, an important issue is the electronic signature of the patient, doctors, and witnesses on informed consent forms.

### Discharge notes

This is a crucial piece of evidence regarding the inpatient treatment of a patient. It is important to give due importance to making a proper discharge summary as this is the summary document that will be kept by the patient which reflects the treatment received. The discharge summary should mirror the case notes of the patient records with a brief summary, relevant investigations, and operative procedures. The dates of admission, discharge, and surgery are useful when the sequence of events is an important issue in litigation later. It is also important to include instructions to be followed by the patient after discharge including dietary advice and date of next follow-up. The doctor can be held negligent if proper instructions are not given regarding the medications to be taken after discharge, physical care that is required, and the need for urgent reporting if an untoward complication happens before the advised time of review. As a urologist, it is common to see patients who are not aware of stents that should have been removed at its appropriate time, though mentioned properly in the discharge summary. The discharge summary should be signed or countersigned by the consultant. A copy of this must be preserved in the case file for future use if required. Discrepancies in the summary given to the patient and what is kept in the hospital records can cause suspicion about tampering with the medical records. These discrepancies should be avoided at all costs as the benefit of this usually goes in favor of the patient.

It is not uncommon to have patients who gets discharged against the advice of the doctor. These patients are also entitled to have a discharge summary about the course of treatment. It is imperative to record the fact that the doctor has advised a course of action with all its implications if not followed. The fact that the patient has understood this and has refused it on his volition should be recorded. This should be signed by the doctor, patient, or relative and duly witnessed. This document has to be retained along with the patient records. It will help the doctor in situations where the patient alleges negligence later.

### Referral notes

Referral notes are an important component of patient records. They should include the date and time of issue, the patient's general condition, cause of reference, and the course of action to be taken. It is wise to keep a duplicate copy of the referral note with the patient's signature. The fact that the patient did not go immediately on reference as advised could be proved by the duplicate copy of the referral note kept by the doctor. This could save a doctor who could be sued for alleged late referral after the patient's condition deteriorated.

## CONFIDENTIALITY OF MEDICAL RECORDS

Medical records can be used as a personal or impersonal document. 1) Personal document - this information is confidential and should not be released without the consent of the patient except in some specific situations. 2) Impersonal document – the record looses its identity as a personal document and patient permission is not required. These records could be used for research purposes. Confidentiality is an important component of the rights of the patient. The hospital is legally bound to maintain the confidentiality of the personal medical records. The patient can claim negligence against the hospital or the doctor for a breach of confidentiality. However, there are certain situations where it is legal for the authorities to give patient information. They are as follows: 1) during referral, 2) when demanded by the court or by the police on a written requisition, 3) when demanded by insurance companies as provided by the Insurance Act when the patient has relinquished his rights on taking the insurance, and 4) when required for specific provisions of Workmen's Compensation cases, Consumer Protection cases, or for Income tax authorities. The maintenance of confidentiality is an important issue in the era of electronic data storage. There should be checks in place so that only those who are authorized can access the patient data.

The impersonal documents have been used for research purposes as the identity of the patient is not revealed. Though the identity of the patient is not revealed, the research team is privy to patient records and a cause of concern about the confidentiality of information. Historically, such research has been exempt from an ethics review and researchers have not been required to obtain informed consent from patients before using their records. Recently, a need has been felt to regulate the use of medical records in research, effectively restricting the manner in which this type of research is conducted. An ethics review is required for using the patient data. However this is not widely followed all over India.

## CATEGORIES OF MEDICAL RECORDS

The different categories of medical records are as follows:

1. Certain records must be given to the patient as a matter of right. Discharge summary, referral notes, and death summary in case of natural death are important documents for the patient. Hence, these have to be given without charge for all including patients who leave against medical advice. The hospital bill cannot be tied up with these sensitive documents that are necessary for continuing patient care. Thus, the above documents cannot be legally refused even when the hospital bill has not been paid.
2. Certain records may be issued after the patient or authorized attendant fulfills the due requirements as stipulated by a hospital. This requires a formal application to the hospital requesting for the records. It is necessary that the hospital bills are cleared and the necessary processing fee has been paid. The documents in this group include copies of inpatient files, records of diagnostic tests, operation notes, videos, medical certificates, and duplicate copies for lost documents. It is important that the duplicate copies should be marked appropriately. It is not unusual for an unscrupulous patient to use it for multiple insurance claims without the knowledge of the doctor.
3. Certain records cannot be given to patients without the direction of the Court. The outpatient file, inpatient file, and files of medico-legal cases including autopsy reports cannot be handed over to the patient or relatives without the direction of the Court. But if these medico-legal cases are being referred to another center for management, copies of records could be given. However, X-rays are given only after a written undertaking by the patient or relatives that these will be produced in the Court as and when required.

## MEDICAL COUNCIL OF INDIA GUIDELINES ON MEDICAL RECORDS

The issue of medical record keeping has been addressed in the Medical Council of India Regulations 2002 guidelines answering many questions regarding medical records. The important issues that have been addressed are as follows:

1. Maintain indoor records in a standard proforma for 3 years from commencement of treatment (Section 1.3.1 and Appendix 3).
2. Request for medical records by patient or authorized attendant should be acknowledged and documents issued within 72 hours (Section 1.3.2).
3. Maintain a register of certificates with the full details of medical certificates issued with at least one identification mark of the patient and his signature (Section 1.3.3).
4. Efforts should be made to computerize medical records for quick retrieval (Section 1.3.4).

## HOW LONG SHOULD MEDICAL RECORDS BE PRESERVED?

There are no definite guidelines in India regarding how long to retain medical records. The hospitals follow their own pattern retaining the records for varied periods of time. Under the provisions of the Limitation Act 1963 and Section 24A of the Consumer Protection Act 1986, which dictates the time within which a complaint has to be filed, it is advisable to maintain records for 2 years for outpatient records and 3 years for inpatient and surgical cases. However the provisions of the Consumer Protection Act allows for condoning the delay in appropriate cases. This means that the records may be needed even after 3 years. It is important to note that in pediatric cases a medical negligence case can be filed by the child after aquiring the age of majority. The Medical Council of India guidelines also insist on preserving the inpatient records in a standard proforma for 3 years from the commencement of treatment. The records that are the subject of medico-legal cases should be maintained until the final disposal of the case even though only a complaint or notice is received. It is necessary that the Government frames guidelines for the duration for which medical records are preserved by the hospitals so that hospitals are protected from unnecessary litigation in issues of medical records.

The provisions of specific Acts like the Pre Conception Prenatal Diagnostic Test Act, 1994 (PNDT), Environmental Protection Act, etc. necessitate proper maintenance of records that have to be retained for periods as specified in the Act. Section 29 of the PNDT Act, 1994 requires that all the documents be maintained for a period of 2 years or until the disposal of the proceedings. The PNDT Rules, 1996 requires that when the records are maintained on a computer, a printed copy of the record should be preserved after authentication by the person responsible for such record.

## OWNERSHIP OF MEDICAL RECORDS

An important issue of dispute between the patient and the treating hospital is about the ownership of the medical records. By and large medical records are the property of the hospitals and it is the responsibility of the hospitals to maintain it properly. The hospitals and the doctors have to be careful with medical records as these can be stolen, manipulated, and misused for malafide reasons by any interested parties. Hence, the records should be in safe custody. It is the primary responsibility of the hospital to maintain and produce patient records on demand by the patient or appropriate judicial bodies. However, it is the primary duty of the treating doctor to see that all the documents with regard to management are written properly and signed. An unsigned medical record has no legal validity. The patient or their legal heirs can ask for copies of the treatment records that have to be provided within 72 hours. The hospitals can charge a reasonable amount for the administrative purposes including photocopying the documents. Failure to provide medical records to patients on proper demand will amount to deficiency in service and negligence.

## SUMMONING MEDICAL RECORDS BY COURTS

Medical records are acceptable as per Section 3 of the Indian Evidence Act, 1872 amended in 1961 in a court of law. These are considered useful evidence by the courts as it is accepted that documentation of facts during the course of treatment of a patient is genuine and unbiased. Medical Records that are written after the discharge or death of a patient do not have any legal value. Erasing of entries is not permitted and is questionable in Court. In the event of correction, the entire line should be scored and rewritten with the date and time.

Medical records are usually summoned in a court of law in the following cases:

1. Criminal cases for proving the nature, timing, and gravity of the injuries. It is considered important evidence to corroborate the nature of the weapon used and the cause of death
2. Road traffic accident cases under the MACT Act for deciding on the amount of compensation
3. Labor courts in relation to the Workmen's Compensation Act
4. Insurance claims to prove the duration of illness and the cause of death
5. Medical negligence cases- these can be in criminal courts when the charge against the doctor is for criminal negligence or under the Consumer Protection Act for deficiency in the doctor's or hospital's care

It is usual to summon a doctor to appear in court to testify and to bring all the medical documents. When the court issues summons for medical records, it has to be honored and respected as it is a constitutional obligation to assist in the administration of justice. The records can also be produced in court by the medical records officer of the hospital. If the doctor is required to be present for giving evidence based on the medical records, he has to be present in the court to give evidence. The court may require these documents to be submitted for which a record is issued by the court. However, if the records are required for continuation of the medical treatment of the patient, copies can be kept by the hospital.

## JUDICIAL DECISIONS IN INDIA ON ISSUES OF MEDICAL RECORDS

There have been many judicial decisions pertaining to medical records from various courts in India and a review of some of the important ones is given in this section.

The National Commission had held that there was no question of negligence for failure to supply the medical records to patients unless there is a legal duty on the hospital to give the records. The alleged hospital had provided a detailed discharge summary to the patient.[1] However, the Bombay High Court held that doctors cannot claim confidentiality when the patient or his relatives demand medical records.[2] With the enforcement of the MCI Regulations, 2002 it has been held without confusion that the patient has a right to claim medical records pertaining to his treatment and the hospitals are under obligation to maintain them and provide them to the patient on request.

The hospital and doctor were guilty of deficiency in service as case records were not produced before the court to refute the allegation of a lack of standard care.[3] The plea of destroying the case sheet as per the general practice of the hospitals appeared to the court as an attempt to suppress certain facts that are likely to be revealed from the case sheet. The opposite party was found negligent as he should have retained the case records until the disposal of the complaint.[4]

Not producing medical records to the patient prevents the complainant from seeking an expert opinion. It is the duty of the person in possession of the medical records to produce it in the court and adverse inference could be drawn for not producing the records.[5] The State Commission held that there was negligence as the case sheet did not contain a proper history, history of prior treatment and investigations, and even the consent papers were missing.[6]

The State Commission held that failure to deliver X-ray films is deficient service. The patient and his attendants were deprived of their right to be informed of the nature of injury sustained.[7] The State Commission disbelieved the evidence of the surgeon because only photocopies were produced to substantiate the evidence without any plausible explanation regarding the absence of the original.[8]

The allegation of not informing the possibility of vocal cord palsy was negated by the detailed written consent that showed that it was explained properly and consented.[9] The allegation of the patient regarding negligence of the doctor was rejected.

The allegation of tampering with the operation notes was negated by the State Commission in a case of intraoperative death as the complainant could not prove the allegation.[10]

The hospital was held vicariously liable for the negligent action of the doctor on the basis of the bill showing the professional fees of the doctor and the discharge certificate under the letterhead of the hospital signed by the doctor.[11] The State Commission held negligence on the basis of the records, which seemed to be manipulated.[12] Issues of tampering of medical records need detailed examination in a civil court rather that in Consumer Court.[13] The National Commission in another case held that the hospital was guilty of negligence on the ground that the name of the anesthetist was not mentioned in the operation notes though anesthesia was administered by two anesthetists. There were two progress cards about the same patient on two separate papers that were produced in court.[14]

Not maintaining confidentiality of patient information can be an issue of medical negligence. The HIV status of a patient was known to others without the consent of the patient.[15]
